# Optical design and performance of the biological small-angle X-ray scattering beamline at the Taiwan Photon Source

**DOI:** 10.1107/S1600577521009565

**Published:** 2021-10-18

**Authors:** D.-G. Liu, C.-H. Chang, L.-C. Chiang, M.-H. Lee, C.-F. Chang, C.-Y. Lin, C.-C. Liang, T.-H. Lee, S.-W. Lin, C.-Y. Liu, C.-S. Hwang, J.-C. Huang, C.-K. Kuan, H.-S. Wang, Y.-C. Liu, F.-H. Tseng, J.-Y. Chuang, W.-R. Liao, H.-C. Li, C.-J. Su, K.-F. Liao, Y.-Q. Yeh, O. Shih, W.-R. Wu, C.-A. Wang, U. Jeng

**Affiliations:** a National Synchrotron Radiation Research Center, Hsinchu Science Park, Hsinchu 30076, Taiwan; b Department of Chemical Engineering, National Tsing Hua University, Hsinchu 30013, Taiwan

**Keywords:** BioSAXS, high flux, ultra-SAXS, microbeam, beamline optics

## Abstract

The optical design and performance of the recently opened 13A biological small-angle X-ray scattering (SAXS) beamline at the 3.0 GeV Taiwan Photon Source of the National Synchrotron Radiation Research Center are reported. The beamline is designed for studies of biological structures and kinetics in a wide range of length and time scales, from angstrom to micrometre and from microsecond to minutes. A 4 m IU24 undulator of the beamline provides high-flux X-rays in the energy range 4.0–23.0 keV. MoB_4_C double-multilayer and Si(111) double-crystal monochromators (DMM/DCM) are combined on the same rotating platform for a smooth rotation transition from a high-flux beam of ∼4 × 10^14^ photons s^−1^ to a high-energy-resolution beam of Δ*E*/*E* ≃ 1.5 × 10^−4^; both modes share a constant beam exit. With a set of Kirkpatrick–Baez (KB) mirrors, the X-ray beam is focused to the farthest SAXS detector position, 52 m from the source. A downstream four-bounce crystal collimator, comprising two sets of Si(311) double crystals arranged in a dispersive configuration, optionally collimate the DCM (vertically diffracted) beam in the horizontal direction for ultra-SAXS with a minimum scattering vector *q* down to 0.0004 Å^−1^, which allows resolving ordered *d*-spacing up to 1 µm. A microbeam, of 10–50 µm beam size, is tailored by a combined set of high-heat-load slits followed by micrometre-precision slits situated at the front-end 15.5 m position. The second set of KB mirrors then focus the beam to the 40 m sample position, with a demagnification ratio of ∼1.5. A detecting system comprising two in-vacuum X-ray pixel detectors is installed to perform synchronized small- and wide-angle X-ray scattering data collections. The observed beamline performance proves the feasibility of having compound features of high flux, microbeam and ultra-SAXS in one beamline.

## Abstract 2

The optical design and performance of the BioSAXS beamline at the Taiwan Photon Source are reported

## 1. Introduction

Synchrotron small-angle X-ray scattering (SAXS) beamlines often particularly emphasize on, for instance, biological SAXS (BioSAXS), grazing-incidence SAXS or ultra-SAXS (USAXS), according to the needs of local user communities. Fast user turnover and result publishing are common characteristics of most synchrotron SAXS beamlines. In the past decades, rapid growth in synchrotron flux has made particularly impressive progress on solution SAXS applications. Further facilitated by versatile and mature public SAXS data analysis software packages (Petoukhov *et al.*, 2012 ▸), SAXS beamlines for protein solution structures have become increasingly popular and are deployed in many synchrotron facilities worldwide (Cowieson *et al.*, 2020 ▸; Li *et al.*, 2016 ▸; Blanchet *et al.*, 2015 ▸).

Previously at the National Synchrotron Radiation Research Center (NSRRC), SAXS activities were initiated in 2002 at the 17B1 wiggler beamline of the synchrotron Taiwan Light Source (TLS) 1.5 GeV storage ring (Hsu *et al.* 2005 ▸). These activities were carried further to the 01B super-bending beamline with an expansion to simultaneous small- and wide-angle X-ray scattering (SAXS-WAXS), using two Gabriel gas-type linear detectors of length 200 mm (Lai *et al.*, 2005 ▸). After that, a SAXS-WAXS endstation was established at 17B3 beamline in 2006, equipped with a Gabriel-type area detector (200 mm by 200 mm) for SAXS and two linear gas detectors for WAXS (Lai *et al.*, 2006 ▸); the gas-type area detector was later upgraded to a MAR165CCD area detector. In 2009, the dedicated NSRRC SAXS beamline 23A was launched (Liu *et al.*, 2009 ▸; Jeng *et al.*, 2010 ▸) to serve the fast expanding SAXS user community, significantly associated with nanostructural research of block copolymers and nanocomposites. Later, the MAR165CCD detector was replaced by a Pilatus 1MF area detector of frame rate 133 Hz (Dectris) for SAXS; the two linear gas detectors were upgraded to two flat-panel detectors CMOS C9827DK and C10158 DK (Hamamatsu) and three Mythen linear detectors of 472 Hz frame rate (Dectris), packed in a row, for WAXS. In 2016, the 3.0 GeV Taiwan Photon Source (TPS) with high brilliance and low emittance (1.6 nm rad) joined operation with the TLS at NSRRC (Horiuchi, 2015 ▸), with a steadily increased storage electron beam current from 300 mA in 2016 to 450 mA in 2021. At the port of TPS 13A, an advanced BioSAXS beamline has been jointly developed by NSRRC and Academia Sinica to catch up with the trend research of SAXS on protein solution structures.

The TPS 13A BioSAXS beamline aims to provide high flux and synchronized, time-resolved SAXS-WAXS for studies of biomacromolecular solution structures over a wide range of length and time scales. The beamline also offers USAXS for studies of hierarchical structures of bio-machinery assemblies in solution, gel or condensed forms. Anomalous SAXS/WAXS for metal or mineral distributions and compositions in organelle or drug carriers can also be performed with a wide X-ray energy range covering the calcium *K*-edge. The beamline applications extend to microbeam SAXS/WAXS for correlated crystal and nano­structural mappings in natural fibril tissues and synthetic biomaterials under tailored environmental controls. The optical designs and performance of the new TPS 13A BioSAXS beamline are detailed below.

## 2. Result and discussion

### 2.1. Photon source

**Figure 1:**
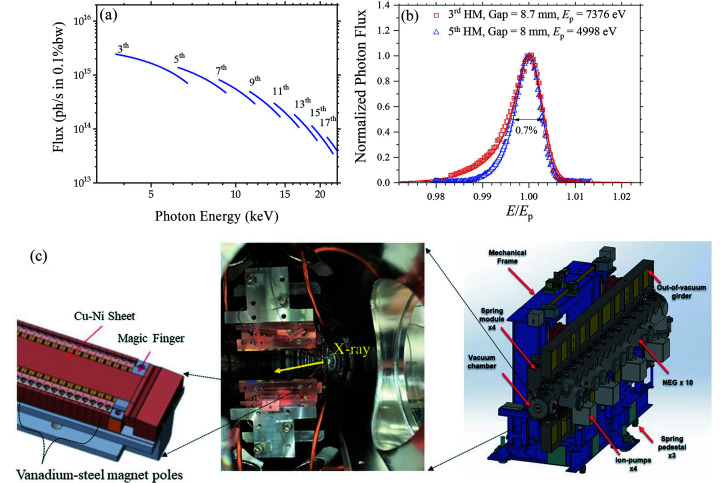
(*a*) Calculated flux profiles of the harmonic modes (3rd to 17th) of IU24, with the parameters shown in Table 1 ▸. (*b*) Measured and normalized flux spectra of the 3rd and 5th harmonic modes of the undulator IU24 at gaps of 8.0 and 8.7 mm, with flux peaked at 7376 eV and 4998 eV, respectively; the data are fitted (solid curves) using the software *Spectra*. (*c*) The 4 m IU24 (right-hand side, with NEG representing for non-evaporable getter pump) comprises up and down vanadium-steel magnet poles (middle) that are sandwiched by blocks of NdFeB alloy. The gap magnet surfaces are covered with Cu-Ni sheets (left).

The BioSAXS beamline uses the TPS port 13A, having a straight section of 7.0 m electron beam path, in which space a 4 m in-vacuum undulator (IU24) (consisting of 168 magnets arranged in a period length of 24 mm) was installed (Fig. 1 ▸). With the 3.0 GeV electron beam and 500 mA beam current of TPS, IU24 provides X-rays in the energy range 4.0–23.0 keV, covering the *K*-edges of calcium (4038 eV) to ruthenium (22117 eV). The effective magnetic peak field *B*_eff_ measured is 0.91 T, with 0.3% error in field uniformity; a maximum effective deflection parameter *K* = 2.03 was derived at 6.8 mm, the designed minimum gap size of IU24. The key parameters of the IU24 are summarized in Table 1 ▸. Fig. 1 ▸(*b*) shows the measured and normalized flux spectra of the third and fifth harmonic modes of IU24 at gap 8.0 and 8.7 mm, having peak fluxes at 7376 eV and 4998 eV, respectively. The spectra are fitted well using the software *SPECTRA* (Tanaka & Kitamura, 2001 ▸) with input parameters of 3.0 GeV electron beam energy (600 bunches), 30 mA beam current, and 518.4 m circumference of the TPS storage ring. The jointly fitted energy spread of 0.11% of the corresponding 3 GeV electron beam matches closely to the design value of 0.088%.

### 2.2. Front-end

**Figure 2:**
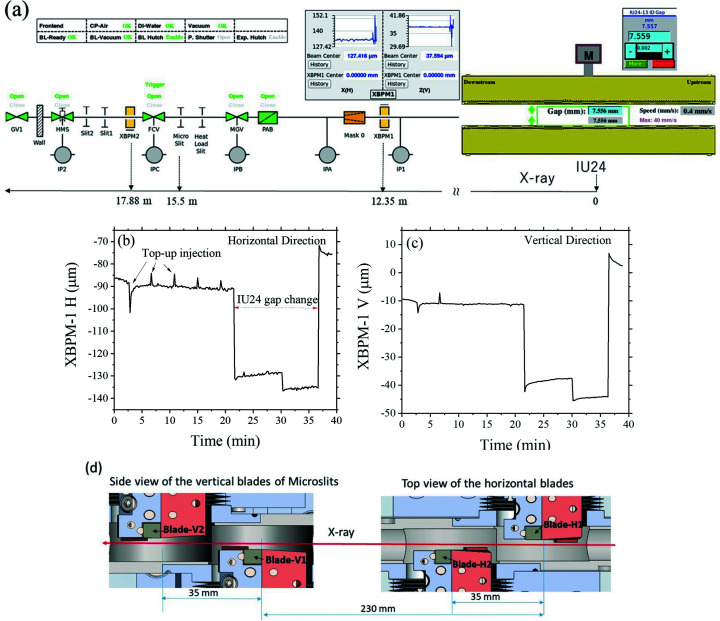
(*a*) Front-end layout of the TPS 13A beamline, featured with the X-ray beam position monitors 1 and 2 (XBPM1 and XBPM2), high-heat-load slits, and microslits. Panels (*b*) and (*c*) are typical beam positions traced in the horizontal and vertical directions by XBPM-1 with 1 µm resolution. Periodic beam-position fluctuations of 5–30 µm correspond to the TPS top-up injections of period 4 min. Also shown in the same time interval is a relatively large beam-position shifting during an occasional change of the IU24 gap. (*d*) Schematics of the microslits, comprising four independent tungsten blades, H1, H2, V1 and V2, separated closely along the beam path for better heat dissipation through individual contacts with copper blocks (colored in orange). The two water-cooled slits of slit1 and slit2 after XBPM2 are optionally used to reduce stray irradiation, hence heat-load on the downstream DCM/DMM. The abbreviations used in (*a*) are PAB (photon absorber), MGV (metal gate valve), FCV (fast closing valve), DI water (deionized water), CP-Air (compressed air pressure), XBPM (X-ray photon beam position monitor), HMS (heavy metal shutter), IPA-C and IP# (ion pumps) and GV (gate valve).

The front-end zone of the beamline outlined in Fig. 2 ▸ comprises mainly two X-ray beam-position monitors (XBPM1 and XBPM2) and the high-heat-load slits followed closely by microslits. Each of the XBPMs consists of four CVD diamond blades with multi-metal layer coating for heat dissipation, as detailed previously (Chuang *et al.*, 2020 ▸). XBPM1, positioned at 12.35 m, was calibrated via the correlated changes of the diamond blade currents (induced upon beam irradiation) measured over the position scanning of the XBPM in the horizontal and vertical directions, respectively (Chuang *et al.*, 2020 ▸). The calibration procedures were further repeated at different IU24 gap sizes (from 7.0 mm to 10.5 mm, with a step of 0.5 mm). The established relationship of the blade-current changes with the XBPM1 positional change (with respect to the beam position) is further parametrized with the changes of the IU24 gap for a compound correlation. The hence calibrated XBPM-1 could report sensitively small beam position changes of a few micrometres during the top-up mode injection of the 3.0 GeV electron beam. Significant positional fluctuations during the IU24 gap change for different X-ray energy could also be observed [Figs. 2 ▸(*b*) and 2(*c*)]. The water-cooled high-heat-load slits comprise two squared frames. The two corners of the squared frames with sharp edges are used to form an aperture down to 50 µm size, to reduce the beam size of the strong white beam in microbeam applications; this is followed by a second set of high-precision microslits of four independent tungsten blades to further confine the beam down to 1–50 µm [*cf*. Fig. 2 ▸(*d*)]. Both the high-head-load slits and microslits are framed with invar materials for better thermal stability and positioned on one large piece of granite to reduce environmental vibrations. We note that these microslits can be varied during beamline operation, under an interlock feedback control to prevent the high-precision microslits from over-radiation due to mismoves.

### 2.3. Beamline optics

**Figure 3:**
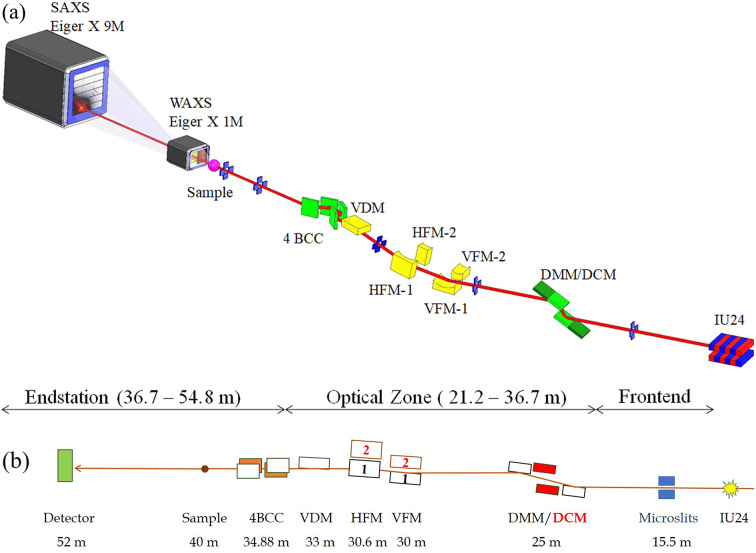
(*a*) 3D drawing and (*b*) side view of the primary optical components and beam path of the TPS 13A BioSAXS beamline. Starting from the right are the undulator IU24, microslits, DMM/DCM, two parallel sets of vertical/horizontal KB focusing mirrors (VFM-1/-2 and HFM-1/-2), vertical deflecting mirror (VDM) and four-bounce double-crystal collimator (4BCC). Located in the endstation zone are the sample stage and the detecting system comprising Eiger X 1M for WAXS and Eiger X 9M for SAXS data collections.

There are four major operation modes of the new beamline, including (1) the high-flux mode for biomacromolecular solution structures and structural kinetics on a microsecond time scale, (2) USAXS mode for resolving hierarchical structures of bio-machinery assemblies of ordered structures up to ∼1 µm, (3) anomalous SAXS (ASAXS) mode for metal or mineral distributions in bio-structures such as liposome drug-carriers, and (4) microbeam SAXS/WAXS for structural mapping of natural/synthetic tissues or textures. The corresponding beamline optics are designed to cover the needs with easy operations of the four modes (Fig. 3 ▸). The major optical components include integrated double-crystal/multilayer monochromators (DMM/DCM) for alternative high-energy-resolution mode (Δ*E*/*E* = 1.5 × 10^−4^) and high-flux mode (Δ*E*/*E* = 8.0 × 10^−3^). The optical system is continued with two parallel sets of beam focusing systems; each comprises a vertical focusing mirror (VFM) facing upward and a horizontal focusing mirror (HFM) facing inward (relative to the storage ring). The focused beam is then leveled by a vertical deflection mirror (VDM), and selectively collimated in the horizontal direction by a downstream four-bounce crystal collimator (4BCC) for USAXS. The corresponding optical systems are detailed further below. We emphasize the largely overlapped beam paths of these characteristic operation modes that allow easy conversions among the operation modes via moving the relevant optical components in and out of the beam path. The corresponding feature parameters of the operation modes are summarized in Table 2 ▸.

#### 2.3.1. DMM/DCM

**Figure 4:**
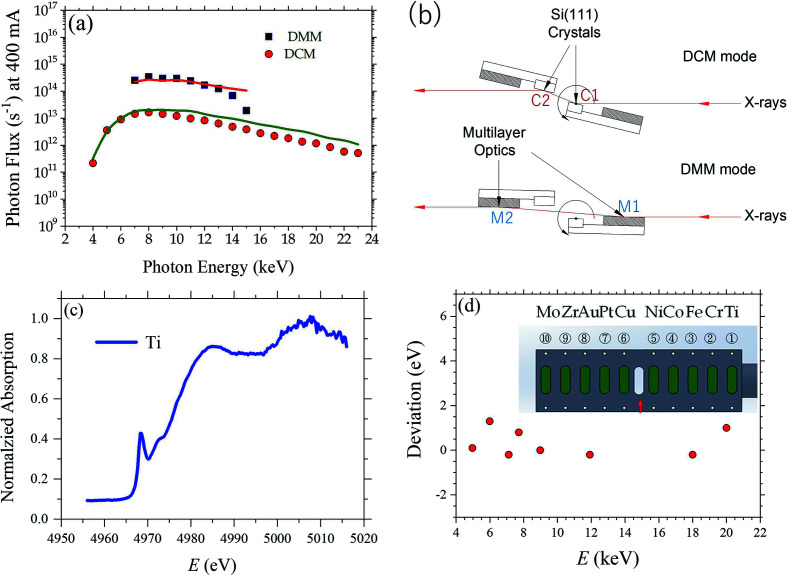
(*a*) DMM/DCM flux spectra measured at the sample position (40 m from the IU24), with an electron beam current of 400 mA. The spectra are fitted largely well with the calculated spectra (solid curves). (*b*) Schematic view of the dual monochromators DMM/DCM, with the rotating center of the whole system situated at the center of the first Si(111) crystal. The rotation range of the DMM/DCM platform from 0.965° to 2.068° is for a 15–7 keV beam with DMM, and 4.93° to 29.62° for 23–4 keV with DCM. (*c*) A typical absorption spectrum of a standard Ti foil taken with the DCM. (*d*) Deviation of the DCM beam energy after calibration with the ten in-vacuum standard foils (inset) deployed downstream of the DMM/DCM.

Following a previous design (Liu *et al.*, 2009 ▸), we integrate Mo/B_4_C double-multilayer and Si(111) double-crystal monochromators (DMM/DCM) on one rotating platform [Fig. 4 ▸(*b*)] (see also Table 3 ▸) to provide a fast conversion between the high-flux DMM mode (∼10^14^ photons s^−1^ in 7–15 keV) and the high-energy-resolution (Δ*E*/*E* ≃ 2 × 10^−4^) DCM mode (4–23 keV). The DMM consists of two sets of multilayers; each has 200 bilayers of Mo/B_4_C with a measured mean lamellar spacing of 24.5 Å and a root-mean-squared roughness of 1.8 Å [Fig. S2 of the supporting information (SI)]. A cryogenic cooling system (Research Instruments GmbH), of 2500 W cooling capacity with liquid nitro­gen (LN_2_), is used to dissipate a maximum heat-load of 31.4 W from the DCM crystals or the DMM multilayers after the TPS 13A impinging X-ray beam. The designed cooling power could keep the surfaces of the multilayers at 85 K or the Si(111) crystal surfaces at 102 K, for which the cooling performance could keep the corresponding slope errors (at the beam spots) below ∼1.5 and 4 µrad (calculated values), respectively. LN_2_ is supplied to the beamline from a central-regulated circulating system of NSRRC. Two diamond filters (100 and 200 µm thick) are placed before the DMM/DCM, to selectively attenuate low-energy X-rays for reduced slope errors of the DCM crystals especially when subjected to X-rays with high-angle incidence.

The flux spectra measured at the 40 m sample position with the DMM/DCM mode (without 4BCC) match largely with the calculated fluxes [Fig. 4 ▸(*a*)], despite slightly larger deviations observed in the region of higher X-ray energy. We note that the flux spectra were measured from the sum current of the quadrant-diamond XBPM Civi-2 (situated at the 40 m sample position), following a previous report (Desjardins *et al.*, 2018 ▸). In the flux deduction, all absorptions from the filters and windows along the beam path were taken into account, including the attenuation due to the two diamond filters and the thin metal coatings of Rigi and Civi-2 XBPMs, and the Be windows. The observed deviations in the measured and calculated flux spectra might associate with the higher transmission (hence lower detecting efficiency) of the quadrant diamond XBPM Civi-2 to high-energy X-rays. Further, with Δ*E*/*E* = 0.8% close to the energy width of 0.7% of the X-ray harmonic modes of IU24 [Fig. 1 ▸(*b*)], the DMM provides monochromatic beams in the energy range 7–15 keV, with beam intensity 20–30 times higher than that of the corresponding DCM beam. We note that the energy-dependent X-ray reflectivity of each set of the DMM multilayers (from 58% of 7 keV to 85% of 15 keV) (Fig. S2, SI) affects critically the DMM performance.

The energy resolution of the DCM beam was calibrated with the absorption spectra of ten standard foils that were selectively moved into the beam path (not far from the DMM/DCM). Shown in Fig. 4 ▸(*c*) is a typical absorption spectrum of the beamline Ti foil, revealing an energy resolution of Δ*E*/*E* ≃ 2 × 10^−4^ of the DCM beam. After a systematic energy offset corrected on the basis of the absorption spectra of the ten foils, the DCM could precisely select the beam energy with an accuracy of a couple of eV, as shown in Fig. 4 ▸(*d*) ▸.

#### 2.3.2. Twin focusing system

**Figure 5:**
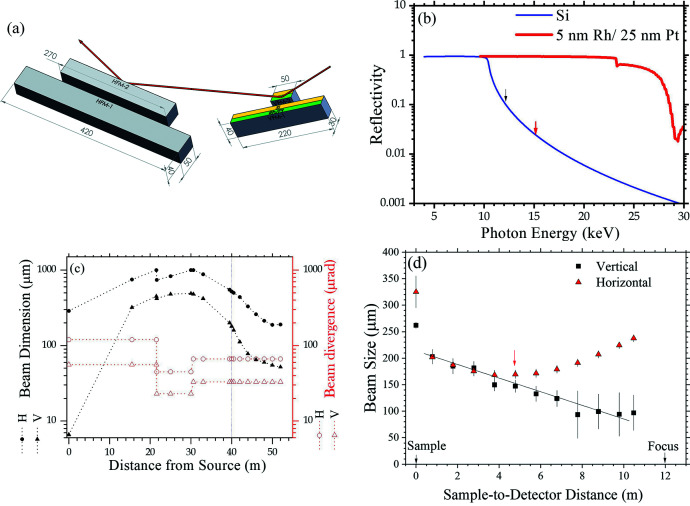
(*a*) The twin focusing system comprising two sets of vertically and horizontally focusing mirrors (VFM and HFM) made of Si crystal blocks with the indicated dimensions (in mm). Half of each mirror surface is coated with Ru/Pt bilayers (colored in green) for enhanced reflectivity of high-energy X-rays; the other half remains bare Si surface (yellow). (*b*) Calculated X-ray reflectivity of the bare Si surface and Pt/Ru-coated surfaces of the focusing mirrors. (*c*) Simulated beam size and divergence (FWHM) over the whole beam path. (*d*) Measured evolution of the beam size from the sample position to the beam focus, using a 10 keV DCM beam and the Eiger X 9M detector. The beam size 280 µm (H) × 50 µm (V) extrapolated (solid line) to the focus position is consistent with the simulated one shown in (*c*). Note that the beam size at the 52 m focus could not be measured due to a length limit (14 m) of the data transmission cables of the Eiger X 9M detector in vacuum.

Fig. 5 ▸(*a*) shows a twin focusing system containing two sets of Kirkpatrick–Baez (KB) focusing mirrors (located side by side for easy swapping) to selectively focus the beam (with 3 mrad incidence) either to the farthest detector position (detector-focusing) for general SAXS or to the sample position for microbeam SAXS (sample-focusing). These mirrors are made of silicon crystal blocks (manufactured by JTEC Co.) of ∼0.1 µrad slope errors and sub-angstrom roughness. Half-strips of the Si crystal surfaces of the VFM/HFM were coated with a double layer of 25 nm Pt on top of 5 nm Rh for higher X-ray reflectivity with beam energy above 10 keV; the other half-strips of bare silicon surfaces of the KB mirrors are used mainly for lower-energy X-rays of 4–10 keV, to better suppress the third-harmonic X-rays from IU24. Fig. 5 ▸(*b*) shows that the Si surface has fast decayed reflectivity with X-ray energy above 10 keV. High harmonic X-rays (above 12 keV) can be reduced by a factor below 10^−3^ via the three Si-surface reflections from the VFM, HFM and a downstream VDM. The calculated evolutions of beam size and divergence with the detector-focusing optics using a ray-tracing program (Sanchez del Rio *et al.*, 2011 ▸) are shown in Fig. 5 ▸(*c*). Evolution of the beam size after the sample position was measured with the Eiger X 9M detector (Dectris; pixel size 75 µm), as shown in Fig. 5 ▸(*d*). The result indicates a focused beam size at the designed position at ∼52 m from the source in the vertical direction. Nevertheless, the beam size measured in the horizontal direction shows a minimum at 47 m, suggesting a larger beam incidence on the HFM than the design value of 3.0 mrad, leading to a slightly shorter focal length. Fortunately, the beam size is still much smaller than the beamstop (4.0 mm diameter) used, and varies slowly in the detecting range of the SAXS detector; the misfocus of the beam in the high-flux mode operation does not obviously affect SAXS measurements and data quality.

**Figure 6:**
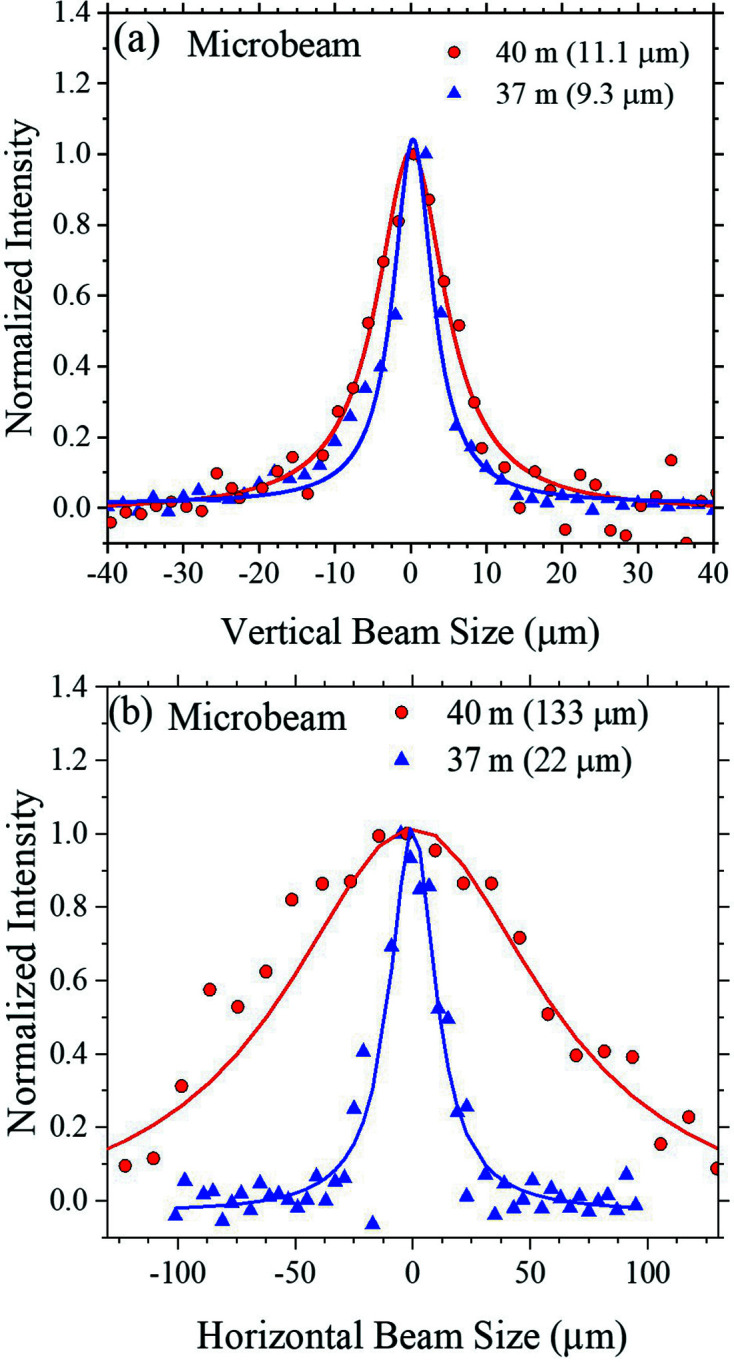
(*a*) Vertical and (*b*) horizontal beam profiles of a 15 keV microbeam with DCM and the sample-focusing KB mirrors measured at 37 m and 40 m positions, using an opening of 10 µm × 10 µm of the microslits. The fitted beam dimensions (FWHM) are indicated in the brackets.

The VFM and HFM of the twin sets of the detector-focusing KB mirrors and the sample-focusing KB mirrors are situated at 30.0 m and 30.6 m positions, respectively, as shown in Fig. 3(*b*) ▸. The sample-focusing KB mirrors are designed to focus the beam defined by the microslits (at 15.5 m position) to the sample (40 m) position with a small demagnification ratio near 1.5 for microbeam applications. With the microslits opening set to 10 µm by 10 µm and a 15 keV beam, the microbeam has a minimum vertical beam size of 11.1 ± 0.8 µm (FWHM) at the 40 m designed focus [Fig. 6 ▸(*a*)] with an optimized flux of ∼1 × 10^10^ photons s^−1^ (without the 4BCC in the beam path); the beam size and flux measured match roughly with the design performance. Nevertheless, the vertical beam size of 9.3 ± 0.4 µm at 37 m is slightly smaller than that at the design focus, at 40 m, suggesting a slightly off-focused beam in the vertical direction. The horizontal beam dimension measured at the 37 m position is 23 µm; however, the beam size measured at the 40 m focus position is 133 µm [Fig. 6 ▸(*b*)]. The misfocus observed might be a result of a significantly larger HFM incident angle used (∼4.0 mrad) compared with the designed 3 mrad incidence; reducing the HFM incident angle toward the designed value however resulted in a significant loss of beam intensity. Improving the horizontal alignment of the central lines of the DCM/DMM and the IU24 source would help resolve the dilemma. We note that such an issue might be circumvented with mirrors of benders for focus tuning, at the expense of the mechanical stability of the mirrors, hence the beam stability.

#### 2.3.3. USAXS with 4BCC

**Figure 7:**
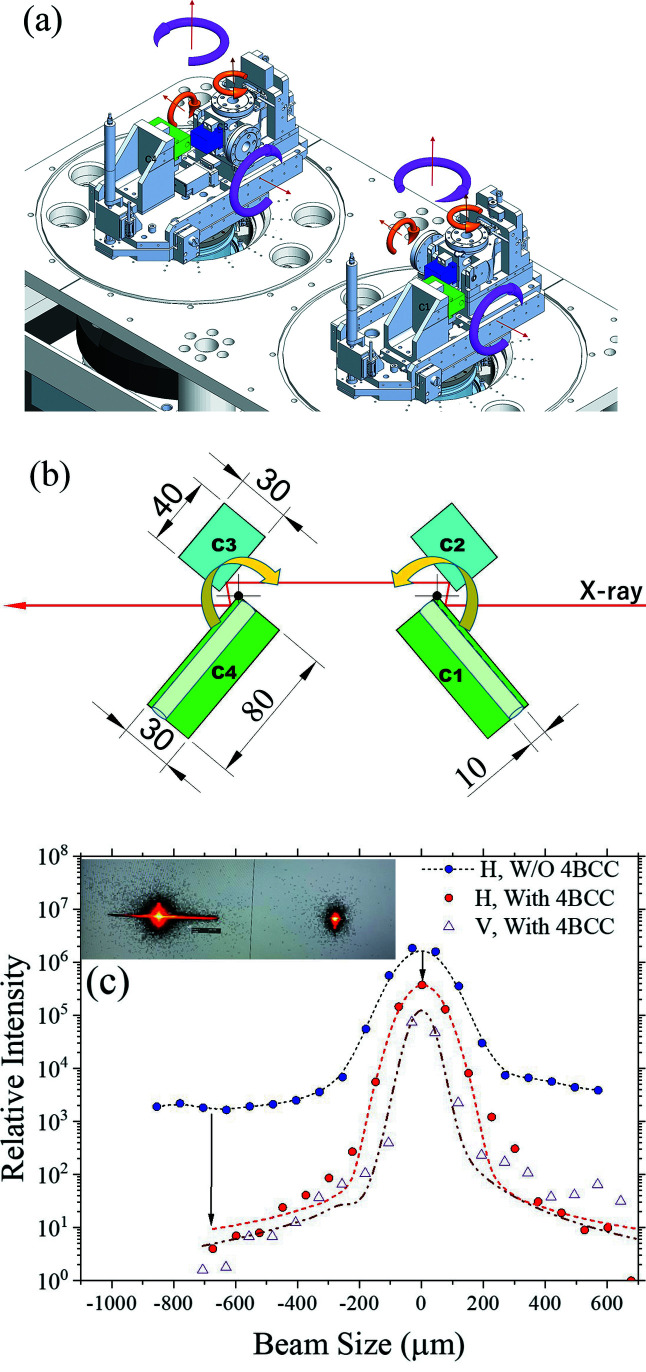
(*a*) 3D drawing of the four-bounce crystal collimator 4BCC (situated at 34.88 m position), comprising two sets of double crystals of Si(111) or Si(311) situated on two large rotating stages for a dispersive configuration. On each rotation stage, the two crystals (C1–C2 or C3–C4) are rotated/titled (*cf.* large arrows) collectively; C2 and C3 crystals further can be rotated/titled (*cf.* small arrows) with respect to the C1 and C4 crystals, respectively. A motorized YAG crystal is installed between C3 and C4 for alignment convenience. The configuration of these two rotation stages can be improved further as detailed in SI. (*b*) An action mode of the 4BCC with the photon beam passing through the four crystals of the 4BCC. With 4BCC rotations (indicated by the arrows), X-rays could bypass 4BCC, through the two 80 mm-long channels in crystals C1 and C4. (*c*) Horizontal and vertical beam profiles of a DCM 8 keV beam measured at 49.5 m near the 52 m focus, without (2D image to the left, inset) and with (2D image to the right, inset) the 4BCC with Si(111). Note that the background scattering intensity near the primary beam is decreased by about two orders of magnitude (long arrow) with the 4BCC, at a cost of a tenfold decay in beam central intensity.

**Figure 8:**
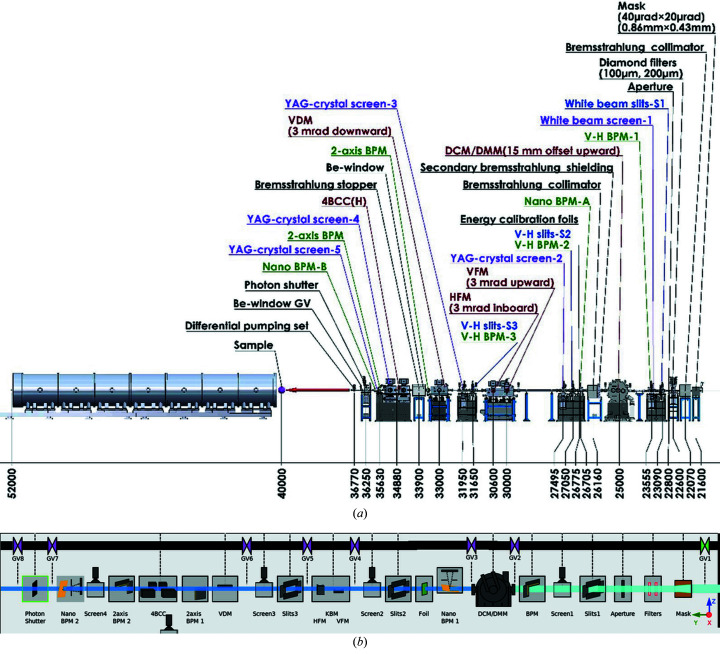
(*a*) Layout of the optical zone (21 m to 37 m) and the endstation (37–55 m) of the TPS 13A beamline. (*b*) The CSS view of the optical zone. The relevant control dialog box would appear upon double-clicking the corresponding icon. VDM, vertical deflection mirror; BPM, beam-position monitor.

For USAXS, a four-bounce crystal collimator (4BCC) [Fig. 7 ▸(*a*)] located downstream is used to further horizontally collimate the monochromatic X-ray beam after the DCM of a vertical diffraction plane. The 4BCC consists of two sets of Si(311) double-crystal collimators in the horizontal diffraction plane arranged in a dispersive configuration [Fig. 8 ▸(*b*)] to significantly decrease the beam divergence to ∼30 µrad. Initially, Si(111) crystals were used for establishing the high-demanding alignment procedure of the 4BCC, as detailed in Fig. S1 (SI). Instead of using channel-cut crystals of common surface roughness of more than 10 Å, the 4BCC of the beamline adopts four independent super-polished crystals with a surface roughness down to ∼2 Å (Sztucki *et al.*, 2019 ▸). The rotation axes of the two double-crystal collimators are located at the upper left corner of the first crystal C1 and the upper right corner of the fourth crystal C4. The parallelism of the paired crystals and the relative orientations of the two sets of double-crystal collimators could be fine-tuned to the 1 µrad parallelism of the level of the channel-cut crystals, using pico-motors (within 1 mrad range) and piezo actuators (100 µrad range). Moreover, there is a 10 mm-diameter tunnel (8 mm below the crystal surface) in both the C1 and C4 crystals, allowing the X-ray beam to bypass the 4BCC diffraction collimation, when the two sets of double-crystal collimators are rotated to be parallel with the X-ray beam [Fig. 7 ▸(*b*)]. Fig. 7 ▸(*c*) shows that the 4BCC with Si(111) (used for a preliminary test) could significantly cut down the 8 keV DCM beam divergence mainly in the horizontal direction for nearly 100 times less background scattering, despite an as-simulated tenfold loss of the X-ray peak intensity to ∼10^11^ photons s^−1^ (Liu *et al.*, 2019 ▸).

#### 2.3.4. Beam diagnostic and control

The quadrant-diamond XBPM-1 (Fig. S3, SI) situated in the front-end zone (before any beamline optical component) is calibrated with different gap-openings of IU24, and can report the beam positions *in situ* with 1 µm resolution. XBPM-1 is also used as a guide in selecting (searching) the local 3 GeV electron beam position and incline angle in IU24, for an optimized beam path to the optical systems of the beamline. Each optical component is equipped with an in-and-out two-position screen made of YAG crystal and a camera readout system for fluorescence beam imaging (50 µm resolution). The beam positions on the screens provide convenient and fast guides in re-alignments and double-checking of the beam path defined by the optical components during conversions of the operation modes or rejections of the 3 GeV electron beam. There are also two FMB-Oxford Nano-XBPMs positioned after the DMM/DCM and 4BCC for beam imaging with sub-micrometre resolution, whose positional information would be used for potential feedback controls of the optical system for beam-position locking.

The beamline adopts the Experimental Physics and Industrial Control System (EPICS) for integrated controls of the hardware and software (Hatje *et al.*, 2007 ▸), including all the motors of the beamline optical components and their corresponding sensing (temperature) and cooling (flow rate) systems. For beamline devices without off-the-shelf EPICS support modules, such as in-house current readout systems of the several quadrant-diamond XBPMs, in-house-made support modules were also collectively implemented into EPICS. Another aspect of the EPICS of the beamline is to serve as a platform in coordinating standalone graphical user interface systems of the beamline add-on devices through the IOC (Input/Output Controller) server program. Communications among local systems are realized via defined process variables (PVs) that can actively propagate out or be accessed timely in the same local networks of the IOC (Chiang *et al.*, 2019 ▸). We have integrated two main clients of PVs to the EPICS of the beamline: (1) the graphical user interface of Control-System Studio (CSS) for visual operations, and (2) a UNIX-based software package *SPEC* (SPEC Control Systems Ltd) for programmable operations of the beamline components and data collection. The CSS provides visual icons, status indicators, interactive dialog inputs for a graphical user interface with controls and status displays of the beamline components. Fig. 8 ▸ shows the major components in the optical zone and the corresponding CSS interface for the components status and visualization controls.

**Figure 9:**
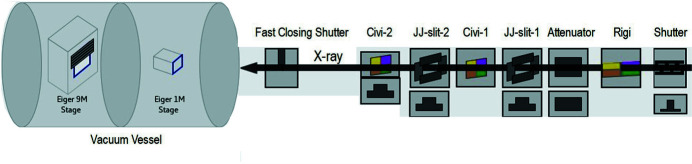
A CSS graphic view (not to scale) of the endstation zone, comprising (from the right) the primary components, including the three XBPMs of Rigi, Civi-1 and Civi-2. The left-hand-side is the detecting system with the two Eiger detectors X 1M and X 9M moving in a large vacuum vessel, 12 m in length and 1.5 m in diameter, for ∼10 m traveling distance along the beam path at a speed of 0.5 m min^−1^. The shortest camera distances are 710 mm and 180 mm for the two detectors, respectively. The Eiger X 9M could move ±120 mm in the lateral and vertical directions, respectively, and is equipped with a 4 mm beamstop.

*SPEC* macro scripts are successfully integrated into the EPICS to perform packaged and interactive operations, including sequential motions of motors, data collection and output for further processing, and input acquirements from different devices via their PVs embedded in the EPICS. For example, the MOVE-E macro could change and optimize the gap of IU24 for a specific X-ray energy, followed by adjusting and optimizing the DCM setting. The macro continues on refining the positions and beam incident angles of VFM and HFM to obtain optimized beam intensity and predefined beam position at the sample position. The powerful automation procedure is executed on the basis of interactive communications with ongoing feedbacks of the beam position and intensity reported by the two endstation XBPMs (Rigi and Civi-1 in Fig. 9 ▸ near the sample position. *SPEC* macro scripts are also applied to the automation of the DMM/DCM mode conversion and the auto-positioning of the beamline slits.

### 2.4. Endstation

The experimental endstation is separated from the optical zone by a lead-embedded wall at 36.5 m. A differential pumping section (720 mm long with 10.7 mm diameter) bridges the ultrahigh vacuum (<10^−7^ torr) of the 4BCC to the first vacuum section in the experimental hutch of higher pressure near 10^−5^–10^−6^ torr. This differential pumping section provides a windowless path for the low-energy beam (mainly 4–5 keV X-rays) to waive beam intensity loss from absorption of a window. Nevertheless, for safety concerns, a UHV gate valve with 250 µm Be window is installed downstream of the 4BCC to selectively isolate the beamline vacuum section from the experiment vacuum section for higher-energy X-rays (of high Be-window transmission).

The first component of the experimental hutch is a vacuum-type fast shutter (8 ms shutter speed), which regulates the exposure of samples to the X-ray beam. Subsequently, an X-ray intensity attenuator, modified from a gas-driven (6 bar) ADC ABS-300, a precision attenuator for hard X-rays (Oxford Co.), can selectively suppress the X-ray beam in 4–23 keV across eight orders of magnitude in intensity. The attenuator could suppress the strong direct beam (up to ∼10^14^ photons s^−1^) for a direct beam image taken with the Eiger X 9M, having a maximum pixel-counting rate of 3 × 10^6^ photons s^−1^. The wide range of attenuation is achieved via ten specifically designed foils made of different thicknesses of Cu, Ta and Al. A user-friendly CSS interface is developed to execute the comparison of an input (target) attenuation factor with all possible attenuation factors calculated from every combination of the ten foils; the calculations are completed through a Python program integrated into EPICS for accessing and sharing information on beam energy and foil transmissions/positions. After confirmation through the CSS interactive dialog box, the selected foils of a best matched attenuation factor are automatically sent into the beam path (*cf*. Fig. 9 ▸). A silicon wafer (10 mm by 10 mm), deposited with a 100 nm-thick silver layer, is positioned downstream inside the attenuator vacuum chamber. The wafer can be selectively moved into the beam path to reflect a laser beam onto the X-ray beam path defined by two sets of JJ X-ray vacuum slits (JJ-slits). Namely, the laser beam path would be tuned to pass through the same two sets of JJ-slits, leading to overlapped X-ray and laser beam paths. This arrangement allows visual guiding on sample positioning for SAXS-WAXS measurements.

The first set of JJ-slits (tungsten carbide, WC, blades with edges glued with Ge crystals) situated at 39.2 m before the sample position and the set of all-tungsten slits (S3) positioned at 31.65 m in the optical zone are used as a major collimation of the X-ray beam. The second set of JJ-slits, of 200 mm mobility along the beam path, can dynamically approach the sample position to minimize the residual air path (source of background scattering) of the beam before entering the detecting chamber. On the two vertical blades of each JJ-slit, four Ta pinholes (of diameters between 0.15 and 0.4 mm) are embedded for the option of pinhole-collimation (versus JJ-slit edge collimation). The pinhole positions to the beam are relatively easily allocated after the slits centering to the beam via automated centering *SPEC* macros.

The beam positions and intensities are monitored online with two four-quadrant-diamond XPBMs: (1) Rigi (quadrant-diamond sensor of 20 µm gap from Dectris) and (2) Civi-1 (3 µm gap, Cividec Co.), which are situated before and after the attenuator, respectively. Another set of XBPMs, Civi-2, was positioned at the sample position for beam size measurements only. These quadrant diamond XPBMs report beam intensity and position with 1 µm resolution, via in-house electric current-reading systems with voltage-to-frequency converters; these readout data are parameterized into PVs of the EPICS, and are shared with CSS for online display and for beam intensity normalization in SAXS-WAXS data processing. Uniquely designed is an integrated detecting system comprising an Eiger X 9M detector for SAXS and an Eiger X 1M detector for WAXS. These two X-ray detectors, of the same pixel size, move independently with multiple degrees of freedom inside a large vacuum vessel of length 12 m and diameter 1.5 m, providing dynamic and quick changes in the detecting configuration. The operation of all components in the endstation zone is integrated into the CSS interface, as shown in Fig. 9 ▸.

### 2.5. Performance tests

**Figure 10:**
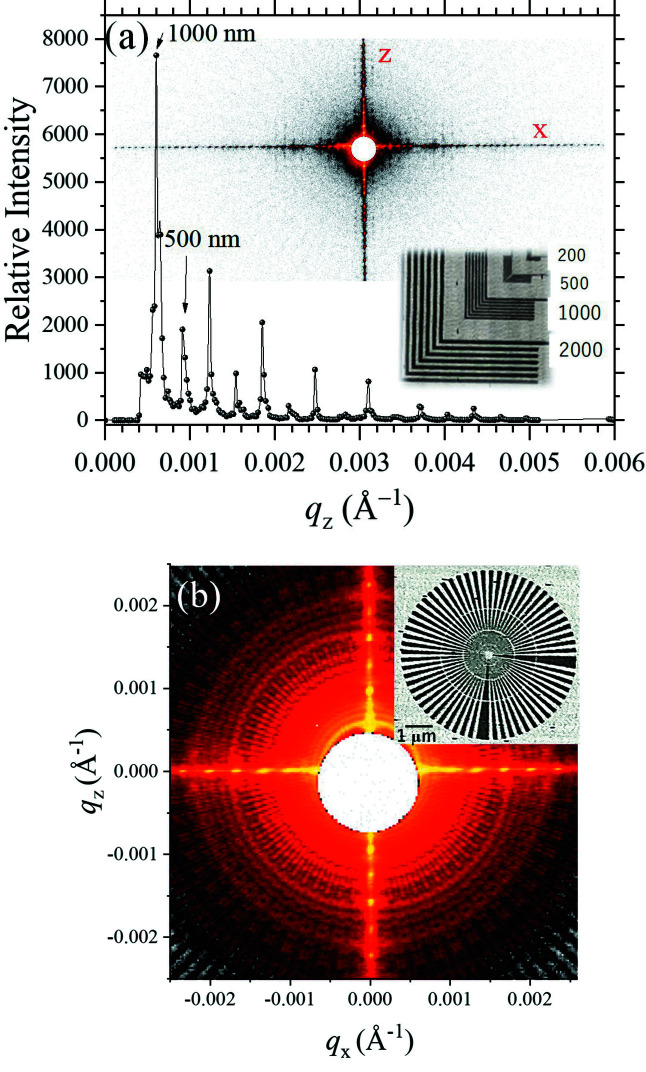
(*a*) USAXS pattern (inset) of an ensemble of gold L-shaped arrays of 2000, 1000, 500 and 200 nm *d*-spacing (inset), measured using a 6 keV beam of DCM with 4BCC. The line-cut profile taken along the *z*-axis reveals the primary peak of the array of 1000 nm spacing located at *q* = 6.3 × 10^−4^ Å^−1^. The minimum detectable *q* is ∼4.0 × 10^−4^ Å^−1^. (*b*) A USAXS pattern of a Siemens star pattern (inset), having 25 nm lines and spaces at the center zone. Note that the fine scattering features are differentiable down to *q* ≃ 0.6 × 10^−4^ Å^−1^.

We tested the USAXS performance using ensemble gold arrays of 2000, 1000, 500 and 200 nm spacing (thickness ∼650 nm) deposited on a silicon nitride (Si_3_N_4_) membrane (Applied Nanotools Inc.), as shown in the inset of Fig. 10 ▸(*a*). With a 6 keV beam of DCM and 4BCC, a sample-to-detector distance of 9.47 m, and a beamstop of 4 mm diameter, the lowest detectable *q* with the Eiger X 9M detector is 4.0 × 10^−4^ Å^−1^, which could resolve clearly the primary peak *q* = 6.28 × 10^−4^ Å^−1^ of the 1000 nm spacing of the gold arrays. Here, *q* is defined by 4πλ^−1^sinθ with X-ray wavelength λ and scattering angle 2θ. Moreover, fine scattering details of a Siemens star pattern fabricated on the same Si_3_N_4_ substrate, of 25 nm lines and spaces at the center zone, could be observed clearly as illustrated in Fig. 10 ▸(*b*), with a discernible *q*-resolution down to 0.6 × 10^−4^ Å^−1^ (matching the design *q*-resolution).

**Figure 11:**
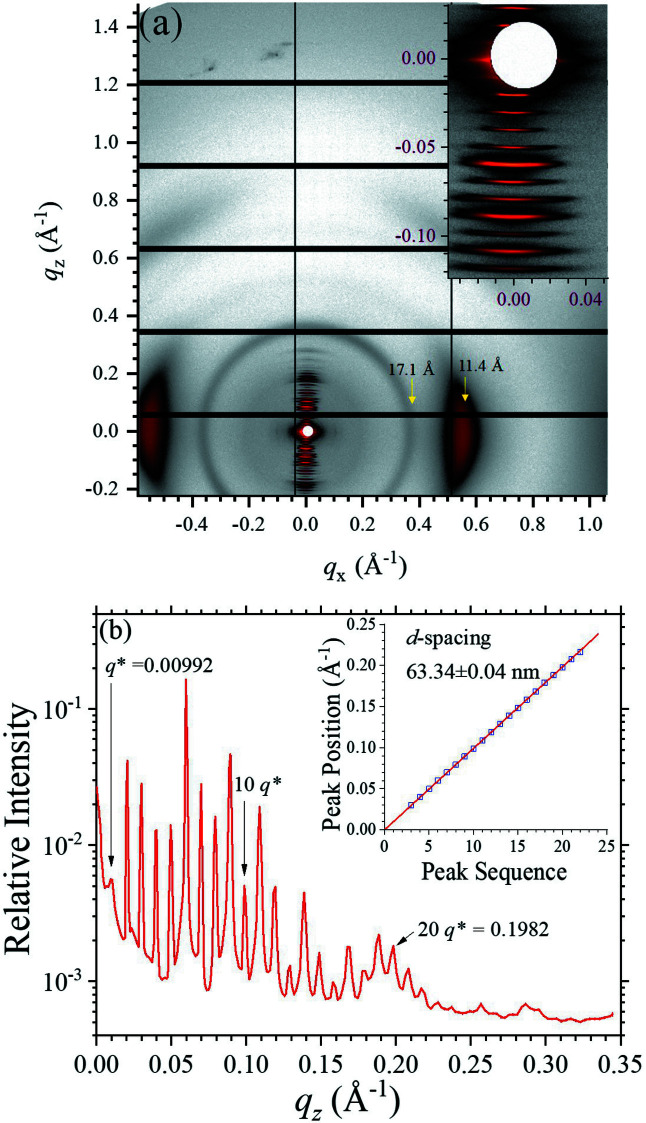
(*a*) Highly oriented 2D pattern taken from a turkey tendon, covering a wide *q*-range as indicated. The inset enlarges the details of the SAXS region for the periodic peaks along the *q*_*z*_ direction. Two prominent arcs located along the *q*_*x*_ axis are selectively marked with the corresponding lateral *d*_L_-spacing of 11.4 Å. (*b*) The intensity line-cut profile extracted along the *q*_*z*_ axis, with the periodic peaks selectively numbered. The inset shows that all the peak positions fall nearly perfectly on the linear regression line, for the *d*-spacing indicated.

Fig. 11 ▸(*a*) demonstrates that the large Eiger X 9M (active area of 233 mm × 245 mm) could cover a wide *q*-range in one imaging for both SAXS and WAXS information, using a sample-to-detector distance of 710 mm and 10 keV beam. With the detecting configuration, a highly asymmetric X-ray scattering pattern was taken from a turkey tendon. In the SAXS region, highly oriented periodic peaks are observed along the *q*_*z*_ axis [Fig. 11 ▸(*a*)], revealing amazingly ordered packing of tropocollagen along the collagen fibril orientation (Maurya *et al.*, 2021 ▸). Deduced from a linear fitting of the observed peak positions [inset of Fig. 11 ▸(*b*)], the first peak position is determined to be 0.00992 Å^−1^ [Fig. 11 ▸(*b*)], corresponding to a *d*-spacing of 63.34 ± 0.04 nm. The corresponding ordered domains size estimated from the peak width is ∼4.0 µm. Moreover, perpendicularly oriented arcs (with respect to the fibril orientation) are observed at *q*_*x*_ = 0.5526 Å^−1^, corresponding to a characteristic Bragg *d*-spacing of *d*_L_ = 11.4 Å. Assuming a 2D-hexagonal packing of the tropocollagen in the cross-sectional direction, we could deduce a lattice constant *a* = 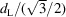 of 13.3 Å, which corresponds to the center-to-center distance of the 2D-hexagonally packed tropocollagen of the turkey tendon (Fang *et al.*, 2012 ▸). Our result demonstrates that orientations and ordered sizes of hierarchically ordered structures could be correlated conveniently and faithfully in one single imaging.

## 3. Conclusion

The TPS 13A biological SAXS beamline has demonstrated its prominent facilities including high flux, USAXS and microbeam. The high flux above 10^14^ photons s^−1^ for studies of structures and kinetics is enabled with the 4 m-long IU24 source and an efficient double-multilayer monochromator; the USAXS is enabled with a long camera length of 10 m and a low X-ray energy beam with small beam divergence and low scattering background, achieved with the combined vertically oriented DCM and horizontally oriented 4BCC; these together allow resolving hierarchically ordered structures up to 1 µm length scale. A microbeam of 10 µm beam size and small beam divergence for structural mappings with simultaneous SAXS/WAXS measurements is achieved with microslits situated in the beamline front-end and a set of dedicated KB mirrors. All these salient features are integrated into the beamline via coordinated optical systems of similar beam paths, which is critical for the multi-mode beamline operations. Graphic CSS and *SPEC* macro scripts are successfully integrated into the EPICS system for complementary and streamline beamline controls, ranging from change of X-ray energy with IU24 to data collection with the two Eiger detectors.

## Supplementary Material

Supporting information Figures S1, S2 and S3. DOI: 10.1107/S1600577521009565/ay5580sup1.pdf

## Figures and Tables

**Table 1:** Features of IU24 at the minimum gap size of 6.8 mm, under an electron beam of 3 GeV and 500 mA of TPS H × V: horizontal × vertical directions. σ: standard deviation of a normal distribution.

Parameter	Designed (measured) value
Period length	24 mm
Number of periods	168
Effective magnetic peak field, *B*_eff_	0.86 T (0.91 T)
Effective deflection parameter, *K*_eff_	1.927 (2.03)
Total magnetic length	4.032 m
Minimum gap size, *g*	6.8 mm (6.8 mm)
Simulated photon beam size (H × V; 1 σ)	120 µm × 4.3 µm @ 4 keV; 120 µm × 2.3 µm @ 24 keV
Simulated photon beam divergence (H × V; 1 σ)	20 µrad × 10.1 µrad @ 4 keV; 19.8 µrad × 9.7 µrad @ 24 keV

**Table 2:** Designed features of the four operational modes of TPS 13A, with beam sizes and divergences in full width at half-maximum (FWHM, 2.35 σ). Note that the IU24 source sizes and divergences shown in Table 1 ▸ are with 1 σ. Microbeam features are calculated with a microslits opening of 10 µm by 10 µm

Operational modes	High flux (DMM)	USAXS Si(111) / Si(311)	ASAXS (DCM)	Microbeam (DCM)
Operation energy range (keV)	7–15	4–15	4–23	4–23
Horizontal demagnification	1.36:1			1.61:1
Vertical demagnification	1.43:1			1.45:1
Beam size H × V (µm @ 52 m)	190 × 52	190 × 36 / 190 × 36	190 × 36	340 × 36 @ 52 m, 7 × 5 @ 40 m
Beam divergence (µrad)	60 × 29	32 × 29 / 12 × 29	60 × 29	70 × 3.8
Energy resolution	0.8%	0.02%		
Flux (photons s^−1^) @ 500 mA	4 × 10^14^	∼10^12^ / ∼10^11^	2 × 10^13^	1 × 10^10^

**Table 3:** Length, width and height (L, W and H) of the crystals C1 and C2 and multi-layers of M1 and M2 of the DMM/DCM shown in Fig. 4 ▸(*b*)

	C1/M1	C2/M2
DCM (L× W × H) (mm)	50 × 40 × 40	110 × 50 × 30
DMM (L × W × H) (mm)	145 × 50 × 30	220 × 50 × 30
