# Supplementary material for: Optical design and performance of the biological small-angle X-ray scattering beamline at the Taiwan Photon Source
Source: J Synchrotron Radiat. 2021 Oct 18;28(Pt 6):1954–65. doi: 10.1107/S1600577521009565 (PMC8570220; doi:10.1107/S1600577521009565)
Supplement: Supplementary file 1 [file s-28-01954-sup1.pdf]

JOURNAL OF SYNCHROTRON RADIATION

Volume 28 (2021)

Supporting information for article:

Optical design and performance of the biological small-angle X-ray scattering beamline at the Taiwan Photon Source

D.-G. Liu, C.-H. Chang, L.-C. Chiang, M.-H. Lee, C.-F. Chang, C.-Y. Lin, C.-C. Liang, T.-H. Lee, S.-W. Lin, C.-Y. Liu, C.-S. Hwang, J.-C. Huang, C.-K. Kuan, H.-S. Wang, Y.-C. Liu, F.-H. Tseng, J.-Y. Chuang, W.-R. Liao, H.-C. Li, C.-J. Su, K.-F. Liao, Y.-Q. Yeh, O. Shih, W.-R. Wu, C.-A. Wang and U. Jeng

S1. Procedure of 4BCC alignment.

The 4BCC alignment procedure includes alignments of the two sets of the double-crystal collimators (DCC) of Si(111) (with the first crystal pair of C1 and C2 and the second crystal pair of C3 and C4), arranged in a dispersive configuration, separately situated on two rotation stages on one big granite. C2 and C3 crystals are mounted on two separate rotation stages. On each the rotation stage, there are picomotors and piezo actuators for coarse and fine adjustments of pitch and roll of the crystal planes. The rotary encoders were mounted on the axes for pitch and roll positional readouts. The piezo stages provide fine adjustment for a crystal rotation angle of 0.1 μrad. A home-made autocollimator was used in the crystal installation for 5 μrad parallelism coarse alignments of the crystal planes of the 2 sets of DCC and their relative orientation. For convenience, a YAG crystal is installed between the C3 and C4 crystals, monitoring the alignment of the C3 crystal to the first set of DCC.

The alignment procedure of the 4BCC is shown in Figure S-1c, including: (1) center the two sets of DCC, to allow X-ray beam passing through the centers of the two through holes of C1 and C4; (2) move out the C3 crystal from the beam path, to adjust C1 and C2 of the first set of DCC using the downstream YAG screen and the X-ray beam position monitor for maximized beam intensity; (3) move in the YAG screen between C3 and C4, and adjust the pitch and roll of C3 for a best YAG illumination intensity; (4) Move out the YAG screen, and adjust C4 for a maximum intensity using the downstream second YAG screen and the beam current of the XBPM. Since C3 and C4 share the same rotation stage, every rotation of second crystal pair requires compensation of counter rotation of C3 using the independent picomotor and piezo actuator for C3; these could maintain the constant relative orientation of C3 to C2 during the alignment procedure of C4 to C3.

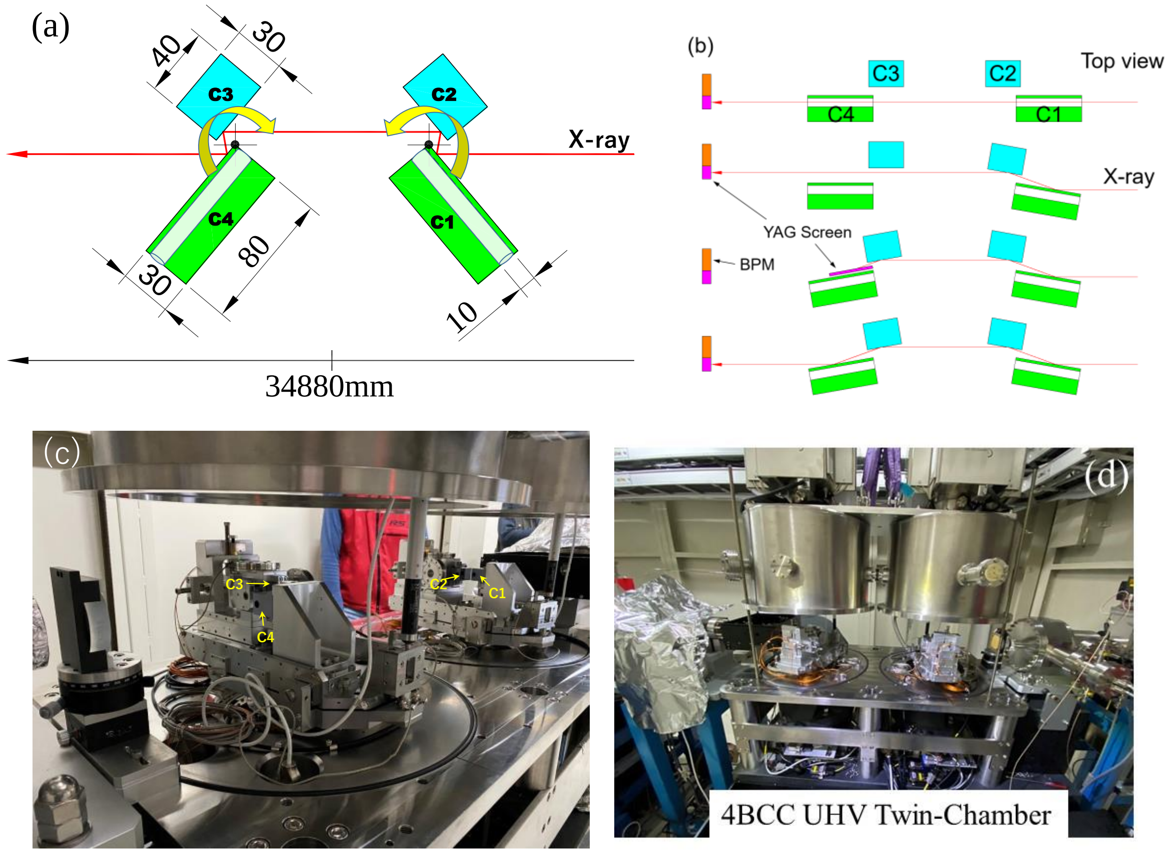

Figure S1 (a) Schematic of the 4BCC. (b) Alignment procedures from the top for C1-C4 alignment, middle for C1-C2 alignment, and down for C3-C4 alignment. (c) The relative positions of C1-C4 inside the 4BCC chamber. (d) The 4BCC chamber consisting of twin 2BCC rotation stages.

S2. Reflectivity of Procedure of 4BCC alignment.

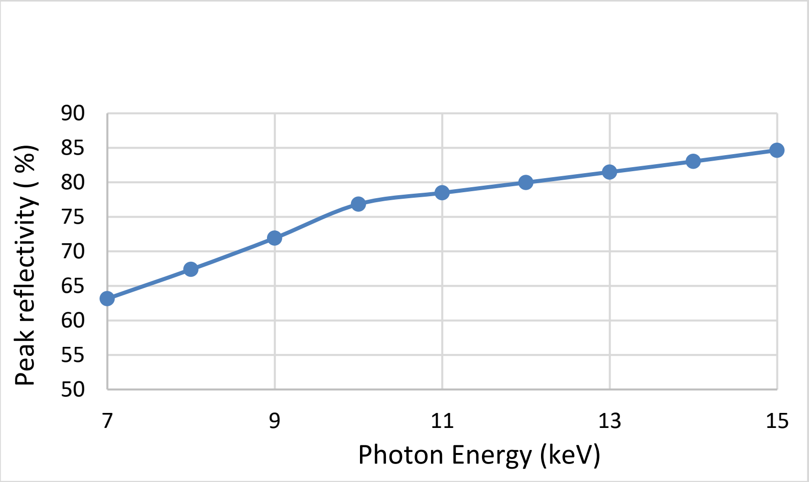

Figure S2 Calculated reflectivity of Mo/B_4C multilayer (provided by Rigaku Co.) as versus of photon energy, based on the following parameters a *d*-spacing = 24.9 Å, number of layers = 200, and root-mean-squared surface roughness σ = 1.8 Å.

S3. 4-quarant diamond XBPM-1 in the frontend zone

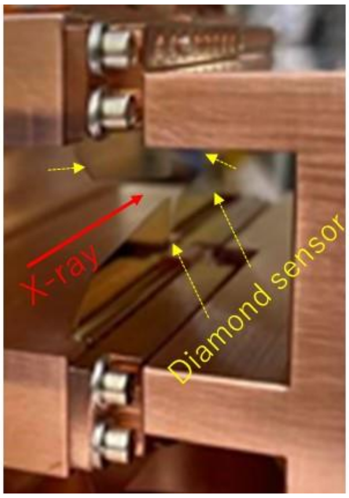

Figure S3 Frontend XBPM-1 with the four-quadrant thin diamond pieces (dotted arrows) fixed on copper cooling blocks.
